# Genomic insights into the mitochondrial architecture of *Sarcandra glabra*: structure, evolution, and functional implications

**DOI:** 10.3389/fpls.2026.1899974

**Published:** 2026-09-21

**Authors:** Yunyi Zhou, Lixiang Yao, Hai Lu, Zheng Xiong, Liying Yu, Chunliu Pan

**Affiliations:** 1 Guangxi Traditional Chinese Medicine (TCM) Resources General Survey and Data Collection Key Laboratory/the Center for Phylogeny and Evolution of Medicinal Plants, National Center for TCM Inheritance and Innovation, Guangxi Botanical Garden of Medicinal Plants, Nanning, China; 2 National Engineering Research Center for Southwest Endangered Medicinal Materials Resources Development, Guangxi Botanical Garden of Medicinal Plants, Nanning, China

**Keywords:** Chloranthaceae, mitochondrial genome, phylogenetic analysis, *Sarcandra glabra*, structural plasticity

## Abstract

**Background:**

*Sarcandra glabra*, an early-diverging angiosperm of the family Chloranthaceae, is extensively used in traditional medicine and has taxonomic importance. However, the research on the structural information of the complete mitochondrial genome of *S*. *glabra* is limited, with no data available for SSR, Ka/Ks ratios, codon analysis, or RNA editing.

**Results:**

In this study, we present the complete mitochondrial genome of *S*. *glabra*, obtained by high-throughput sequencing using Illumina and Nanopore platforms. It enabled the assembly of a multi-branched genome, consisting of a linear chromosome of 1,120,880 bp and a circular chromosome of 192,931 bp. The annotation identified a total of 81 genes, including 39 protein-coding genes, 21 distinct transfer RNAs with multiple gene copies, and 3 ribosomal RNAs, with an overall GC content of 46.03%. The analysis across 9,547 codons revealed a strong preference for A or U ending codons, and serine was found to be the most common amino acid. A total of 524 RNA editing sites were authenticated via RNA-seq, with the *nad5* exhibiting the highest frequency of editing events. Ka/Ks analysis showed that the majority of genes were under purifying selection, while increased ratios in *nad4*, *cob*, and *rpl16* implied instances of adaptive evolution. Extensive potential for chromosomal rearrangement was shown by 399 simple sequence repeats and over 3,585 interspersed repetitions, mostly consisting of short forward or palindromic motifs. Phylogenomic analyses of 21 angiosperm mitochondrial genomes placed *S*. *glabra* within Chloranthaceae, clustering with *Chloranthus spicatus* and forming a well-supported sister relationship with *Hedyosmum orientale*.

**Conclusions:**

This work offers the first comprehensive analysis of the *S*. *glabra* mitochondrial genome, highlighting its structural complexity, dynamic RNA editing, signatures of adaptive evolution, and its phylogenetic position among basal angiosperms.

## Introduction

*Sarcandra glabra* (Chloranthaceae), referred to as Cao Shan Hu in traditional Chinese medicine, is a perennial evergreen shrub found in southern China, Southeast Asia, and some regions of South Asia (**Supplementary Figure S1**). It has been widely used in folk medicine for centuries (Zeng et al., 2021). Ethnopharmacological studies have shown its efficacy in addressing inflammation, viral infections, and cancer-associated ailments (Cao et al., 2012; Li et al., 2016). Phytochemical investigations have identified many secondary metabolites, including flavonoids, coumarins, and phenolic compounds, that enhance its therapeutic effectiveness (Liu et al., 2016).

Beyond its practical use, *S*. *glabra* has taxonomic importance as a member of the Chloranthaceae family, an ancient lineage of angiosperms often positioned at the base of flowering plant phylogeny (Qiu et al., 1999; Zeng et al., 2014). The phylogenetic position of Chloranthaceae has consistently intrigued botanists, as it offers essential insights into the early diversification of angiosperms and the evolution of floral and reproductive characteristics. Several chloroplast genomes of *S*. *glabra* and related species within Chloranthaceae have been sequenced and utilized in phylogenetic analyses. For example, Li et al. revealed a monophyletic Chloranthaceae positioned as sister to the Lilianae (Wang et al., 2020; Li PW. et al., 2025). Han et al. identified that Chloranthaceae was sister to magnoliids based on chloroplast genome data (Han et al., 2018), aligning with phylogenomic analyses using extensive nuclear datasets (Wickett et al., 2014). Moreover, phylogenies derived from nuclear markers such as ITS and low-copy nuclear genes have sometimes produced different topologies. For instance, Qiu et al. positioned the family closer to monocots and eudicots (Qiu et al., 2010). These disparities underscore the difficulties of reconstructing early angiosperm relationships and suggest that different genomic compartments may have conflicting phylogenetic signals owing to incomplete lineage sorting, ancient hybridization, or biased substitution patterns.

Mitochondrial genomes are becoming acknowledged as valuable indicators that complement plastid and nuclear data (Gualberto and Newton, 2017). Chloroplast genomes have generally conserved sequences, but mitochondrial genomes often change differently, providing enhanced precision for reconstructing evolutionary relationships (Zeng et al., 2014). Mitochondria are essential organelles in almost all eukaryotic organisms, playing a central role in energy metabolism, programmed cell death, and the regulation of metabolic signaling pathways (Millar et al., 2011). In plants, mitochondrial genomes have significant complexity relative to those in animals (Burger et al., 2003). Animal mitochondrial genomes are typically compact, circular entities around 16 kb in size, with very comparable gene content. Plant mitochondrial genomes exhibit significant variability in size, ranging from around 66 kb to over 11 Mb. They often possess multipartite structures, undergo frequent recombination, and include substantial repetitive components (Sloan et al., 2012). These unique characteristics of plant mitochondrial genomes make them important for studying genome evolution, intracellular gene transfer, and phylogenetic relationships across plant lineages (Palmer et al., 2000). Despite the mitochondrial genome of *S*. *glabra* being submitted to GenBank, a formal publication offering a thorough analysis has yet to be released. The availability of raw sequences alone cannot replace comprehensive analyses of genome structure, repeat elements, RNA editing sites, and phylogenetic implications. A complete and well-annotated mitochondrial genome, together with comparative and evolutionary analyses, is therefore critical for understanding the molecular evolution of early-diverging angiosperms and for supporting the utilization of *S*. *glabra* as a medicinal resource.

In summary, this study aims to characterize the mitogenomes of *S*. *glabra* by sequencing and assembling its complete mitogenome using Nanopore and Illumina technologies. The objectives were as follows: (1) elucidate the molecular features of *S*. *glabra*; (2) characterize genomic characteristics, including structural organization, repeat elements, and RNA editing sites and (3) compare the *S*. *glabra* mitochondrial genome with those of related angiosperms to identify evolutionary patterns. The findings will contribute to our knowledge of plant mitochondrial genomics of *S*. *glabra*. By integrating genome characterization with comparative and phylogenetic analyses, it also offers novel insights into the evolution of early angiosperms and enhances the molecular resources available for a widely used medicinal plant.

## Materials and methods

### DNA extraction, genome sequencing, and assembly

The leaves of well-grown living *S*. *glabra* were collected from the plant cultivation research base of the Guangxi Botanical Garden of Medicinal Plants (Nanning, Guangxi, China). A voucher specimen from the same plant was taxonomically identified by Prof. Liying Yu and deposited in the Guangxi Chinese Herbal Medicine Herbarium, Guangxi Botanical Garden of Medicinal Plants (herbarium code: GXMG; voucher No.: 45010220250805001LY). High-quality genomic DNA was extracted using a modified CTAB method.

A combination of sequencing platforms was used to generate genomic data. High-molecular-weight DNA was used for long-read sequencing on the Nanopore PromethION platform (Nanodrop Technologies, Wilmington, DE, USA), and 22.50 Gb raw data with average reads size of 10.56 kb were produced in *S*. *glabra.* Raw data were filtered and reprocessed with NanoFilt and NanoPlot inside Nanopack (De Coster et al., 2018). Moreover, libraries with an average fragment length of 350 bp were generated from high-quality DNA using the NexteraXT DNA Library Preparation Kit, and sequencing was performed on the Illumina NovaSeq 6000 platform (Illumina, San Diego, CA, USA), generating 150 bp paired-end reads for genome survey and error correction. 21.83 Gb raw sequence data were produced in *S*. *glabra*. The data were further processed and subjected to quality control using the NGS QC Tool Kit v2.3.3 (Patel and Jain, 2012).

Initially, Nanopore sequencing data were aligned to the reference genome (NCBI Reference Sequence: *S*. *glabra*, OR668929-OR668930) via minimap2 (v2.15-r905). The preliminary assembly was conducted with miniasm (v0.3-r179), then refined with nextPolish (v1.3.1, https://github.com/Nextomics/NextPolish). Subsequently, second-generation Illumina reads were aligned to the corrected assembly using Bowtie2 (v2.3.5.1), and the resultant data were assembled into contigs employing Unicycler (v0.4.8), followed by visualization with Bandage (Wick et al., 2015). Finally, the Nanopore data were realigned with the contigs via minimap2 to verify contig linkages, which were then manually curated to provide the final mitogenome assembly.

The whole mitochondrial genome of *S*. *glabra* was annotated using MITOFY (Alverson et al., 2010) and MFANNOT (Gautheret and Lambert, 2001), via comparisons with established angiosperm mitochondrial genes in the NCBI database (https://blast.ncbi.nlm.nih.gov). The circular map of the mitogenome was shown using the OGDRAW program (Greiner et al., 2019). The mitochondrial genome of *S*. *glabra* has been submitted to GenBank (NCBI) with the accession number: PX754771 and PX754772.

### Repeat sequence analysis

Simple sequence repeats (SSRs) were detected using MISA software (v1.0) with the parameters: 1-10, 2-5, 3-4, 4-3, 5-3, and 6-3. Tandem repetitions were detected using the TRF program (v4.09.linux64). This research used alignment parameters of (2, 7, 7), established a minimum alignment score of 70 for reporting repeats, and set a maximum period size of 2000. Interspersed repetitions were examined via REPuter (https://bibiserv.cebitec.uni-bielefeld.de/reputer/), with a minimum repeat length of 30 bp and an identity criterion of 90% (Hamming distance of 3). The outcomes were shown using Circos v0.69-6.

### Codon usage bias and RNA editing event analysis

Protein-coding sequences (CDS) from the mitochondrial genome were obtained. Sequences less than 300 bp and duplicates were eliminated, retaining only those sequences that started with ATG and concluded with TAA, TAG, or TGA. Relative synonymous codon usage (RSCU) values and the effective number of codons (ENC) were calculated using CodonW v1.4.4 (http://codonw.sourceforge.net). The RSCU heatmap was generated using the RSCU-Plot web tool (https://pcg-lab.shinyapps.io/RSCU-Plot/) (Akrami et al., 2025; Diani Gohar and Soorni, 2025; Hejazi et al., 2025; Soorni and Golchini, 2025), which provides publication-quality visualization. To further characterize codon usage patterns, an ENC-plot was constructed by plotting ENC values against GC3s (the GC content at the third synonymous codon position). In addition, a parity rule 2 (PR2) bias plot was generated by calculating the frequencies of A, U, G, and C at the third codon position of four-codon amino acids, and plotting A/(A + U) against G/(G + C) to evaluate the relative contributions of mutation pressure and natural selection.

RNA editing occurrences were forecasted with the PmtREP tool. To empirically validate the computationally predicted RNA editing sites, corresponding transcriptomic data (NCBI SRA accession: SRR25030271) was utilized. The raw RNA-seq reads were mapped to the mitochondrial protein-coding sequences using Bowtie2 v2.4.1. Subsequently, the alignment results were converted and sorted using SAMtools v1.9. To identify the genuine RNA editing events, BCFtools v1.10.2 was employed to call single nucleotide polymorphisms between the transcriptomic reads and the reference mitochondrial genome. Only the reliable mismatch sites supported by the RNA-seq data were retained as validated RNA editing sites for downstream analyses.

### Mitochondrial–plastid transfers analysis

To ensure the accurate and specimen-specific evaluation of intracellular gene transfers, the complete chloroplast genome of the exact same *S*. *glabra* individual was first *de novo* assembled from our sequencing data using GetOrganelle v1.5.2 and subsequently submitted to GenBank (accession number: PZ769974). Homology searches across the mitochondrial and plastid genomes of *S*. *glabra* were conducted using BLASTn v2.9.0+ with the settings -evalue 1e-5 and -word_size 7. Only transfer events involving pieces above 1,000 bp were deemed relevant for study. The outcomes were visualized using Circos v0.69-6.

### Nucleotide diversity analysis

Homologous gene sequences from several species were aligned with MAFFT (v7.429). Nucleotide diversity (Pi) for each gene was computed using DnaSP6 (v7.0) software, and the findings were shown. The sliding window length was established at 200 bp, with a step size of 100 bp.

### Ka/Ks analysis

Homologous gene pairs were retrieved and aligned for pairwise species comparisons using ParaAT2.0 software with default settings. The Ka and Ks values for each gene pair were computed using KaKs_Calculator v2.0 (https://sourceforge.net/projects/kakscalculator2/), using the YN method for the calculations. The Ka/Ks ratio for each gene pair was later calculated.

### Phylogenetic analysis

To deduce the phylogenetic relationships, 21 representative angiosperm species were chosen. The taxon sampling covered Lauraceae (*Cinnamomum camphora*, *C. chekiangense*, *Machilus pauhoi*, *M. yunnanensis*, *Persea americana*, and *Caryodaphnopsis henryi*), Hernandiaceae (*Hernandia nymphaeifolia*), Magnoliaceae (*Magnolia biondii*, *M. liliiflora*, *M. officinalis*, *M. figo*, and *Liriodendron tulipifera*), Calycanthaceae (*Chimonanthus praecox*), Saururaceae (*Houttuynia cordata* and *Saururus chinensis*), Piperaceae (*Peperomia blanda*), and Chloranthaceae (*S*. *glabra* and *Chloranthus spicatus*), along with *Hedyosmum orientale* representing Chloranthales and *Amborella trichopoda* as the outgroup. The mitochondrial genomes of these species were acquired. OrthoFinder (v2.3.14) was used to find orthologous single-copy genes, including 11 *genes: ccmB, ccmC, ccmFc, cob, cox2, nad4, nad5, rpl16, rps11, rps14*, and *rps3*. The genes were retrieved with Perl scripts and aligned with MAFFT (v7.429). Regions containing alignment gaps were filtered using Gblocks 0.91b. The resulting concatenated sequences were used to create the phylogenetic tree using IQ-TREE v1.6.12, with the optimal nucleotide substitution model determined by ModelFinder, according to the Bayesian Information Criterion (BIC). A maximum likelihood (ML) tree was constructed using 1,000 bootstrap repetitions. Additionally, to evaluate the utility of potential phylogenetic markers, six supplementary ML trees were constructed using the identical analytical pipeline (MAFFT, Gblocks, and IQ-TREE) based on different sequence datasets: all genes, all conserved PCGs, concatenated hypervariable genes (*atp1*, *atp9*, and *nad6*), and individual alignments of *atp1*, *atp9*, and *nad6*.

## Result

### Mitochondrial genome assembly and annotation

We used Illumina and Nanopore sequencing systems to sequence DNA samples of *S*. *glabra* in order to gather basic data for mitogenome assembly. The quality control (QC) results of the mitochondrial sequencing of *S*. *glabra* were evaluated first. For Illumina sequencing, a total of 1.46 billion raw reads and 2.18 billion raw bases were generated. After quality filtering, 1.44 billion clean reads and 2.14 billion clean bases were retained. The Q20 and Q30 ratios were 97.97% and 93.83%, respectively. The proportion of reads that passed filters was 99%, with only a small fraction of reads showing low quality (966,988 reads), excess N content (23,896 reads), or being too short (33,474 reads). For Nanopore sequencing, a total of 2.15 million reads were generated, with 2.05 million reads passing the quality control.

Based on these high-quality sequencing data, the mitogenomes of *S*. *glabra* were assembled and found to have a multi-branched structure, consisting of a linear molecule (Molecule 1) and a circular molecule (Molecule 2), with size of 1,120,880 bp and 192,931 bp, respectively (**Figure 1**). The average sequencing depths for the Illumina and Nanopore data reached 122.0× and 43.1× across the entire mitogenome (**Supplementary Figure S2**). Genome annotation identified 81 genes, including 39 protein-coding genes (PCGs), 21 distinct transfer RNA (tRNA) genes, and 3 ribosomal RNA (rRNA) genes (**Supplementary Table S1**). Of the tRNA genes, 12 had multiple copies, ranging from 2 to 6. Molecule 1 had a GC content of 45.98% and encodes 31 protein-coding sequences with a combined length of 28,359 bp, together with 35 tRNA genes spanning 2,667 bp and two rRNA genes spanning 5,659 bp. The average GC contents of the coding sequences, tRNAs, and rRNAs were 44.94%, 51.29%, and 52.68%, respectively. Molecule 2 exhibited a slightly higher GC content of 46.34% and contained nine protein-coding sequences with a total length of 6,279 bp, four tRNA genes covering 293 bp, and one rRNA gene with a length of 116 bp. The corresponding GC contents of these gene categories were 42.51%, 51.54%, and 52.59%, respectively (**Supplementary Table S2**). The 81 genes were categorized into 13 functional categories, covering essential mitochondrial functions like ATP synthesis, electron transport, cytochrome c biogenesis, ribosomal function, and RNA maturation.

**Figure 1:**
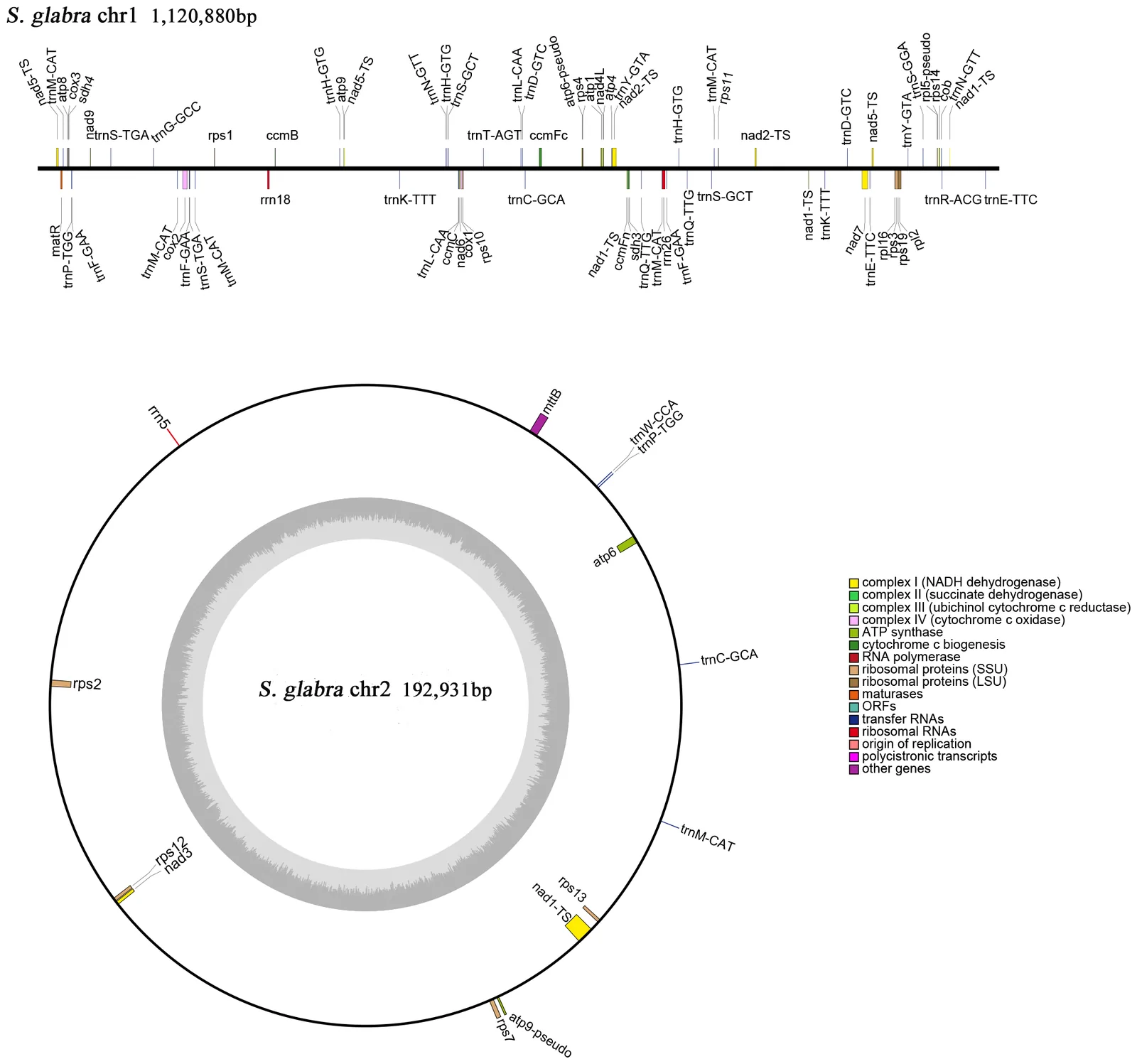
Structure and functional annotation of the *S*. *glabra* mitochondrial genome. *S*. *glabra* mitochondrial genome comprises two chromosomes (chr1 and chr2). Genomic features transcribed clockwise and counter-clockwise are drawn on the inside and out-side of the circle, respectively. The inner circle (dark- and light-gray color) represent GC and AT content. Genes are color-coded according to functional categories. Transfer RNA genes are labeled with their corresponding amino acid codes.

To further investigate the evolutionary driving forces shaping these codon usage biases, an ENC-plot analysis was conducted (**Figure 2B**). The ENC values for the protein-coding genes varied from 42.44 to 61.00, with an average value of 52.68. Notably, the majority of the genes clustered well below the standard expected curve. This distribution indicated that while mutation pressure exists, natural selection plays a more dominant role in driving the codon usage bias of the *S*. *glabra* mitogenome. Furthermore, a PR2-bias plot analysis (**Figure 2C**) revealed an unequal distribution of the four nucleotides at the third codon position. Most genes were distributed in the left half of the plot (G3/(G3+C3) < 0.5), indicating a distinct preference for C over G at the third codon position. This disproportionate use of bases and the deviation from the center strongly corroborated that the combined effects of mutation pressure and natural selection shape the codon usage patterns in this mitochondrial genome.

**Figure 2:**
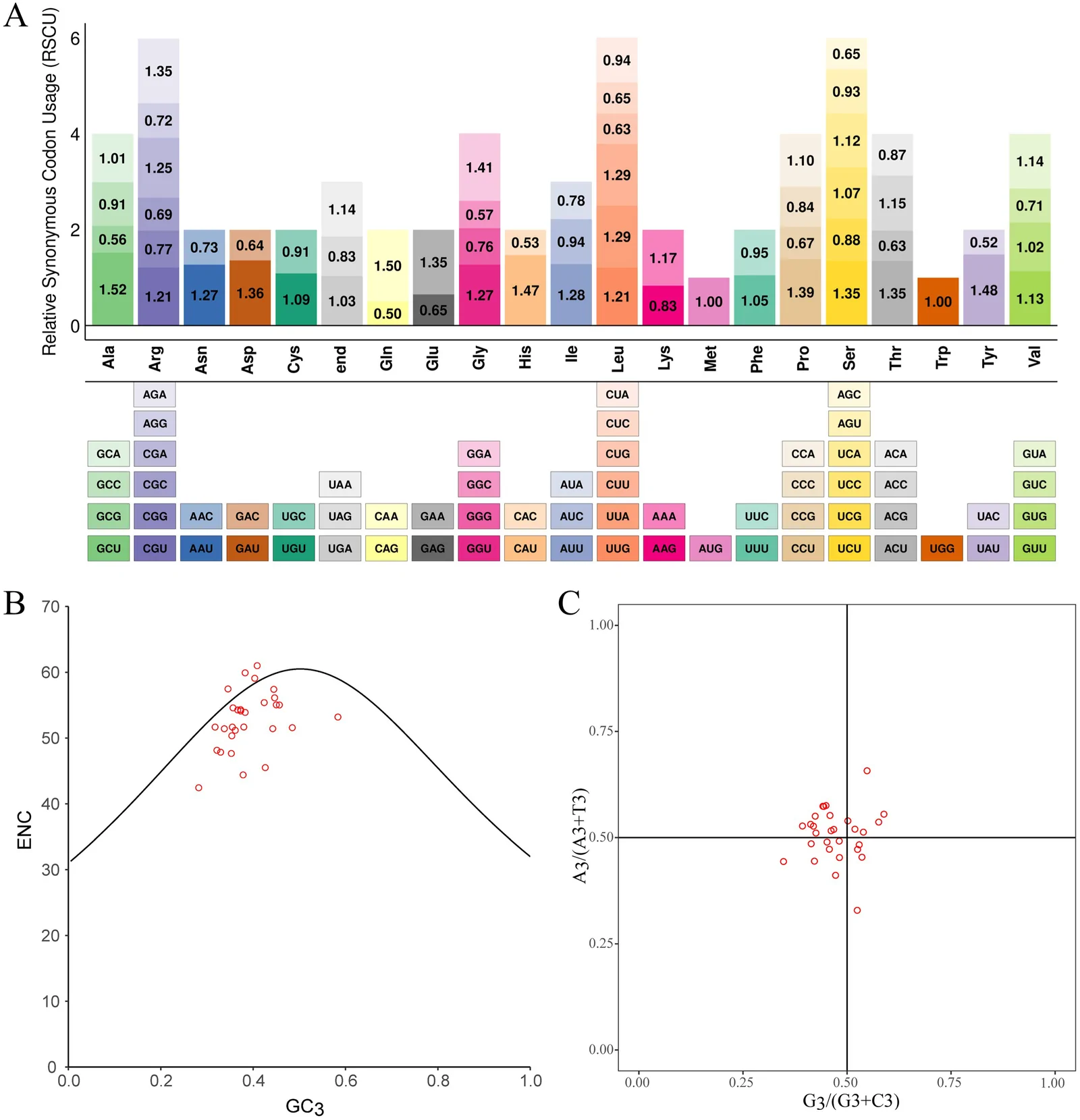
Codon usage patterns and evolutionary driving forces in the *S*. *glabra* mitochondrial genome. **(A)** Relative synonymous codon usage (RSCU) values. Each color-coded block within a bar represents a specific codon, as labeled below the plot. The height of each segment indicates the relative frequency of that codon’s usage compared to equal usage of synonymous codons for the same amino acid. **(B)** The ENC-plot analysis evaluating the influence of mutation pressure versus natural selection. The solid black line represents the expected curve if codon usage is solely driven by mutation pressure. **(C)** The PR2-bias plot illustrating the distribution of four bases at the third codon position.

### Codon usage

Codon usage analysis offers critical insights into the evolutionary pressures influencing plant mitochondrial genomes, especially translational selection, and functions as a valuable resource for comparative genomics and phylogenetic studies. In the present study, codon usage analysis revealed a total of 9,547 codons among the PCGs of the *S*. *glabra* mitochondrial genome (**Supplementary Table S3**). Upon categorizing codons by amino acid, Serine was the most abundant amino acid, encoded by 938 codons. Other often represented amino acids were Leucine (Leu), Arginine (Arg), and Isoleucine (Ile). Amino acids such as Cysteine (Cys) and Tryptophan (Trp) were the least represented, comprising 1.5% and 1.4%, respectively. Codon usage exhibited biased, demonstrating clear preferences for certain synonymous codons (**Figure 2A**). There were 19 out of the 20 amino acids that showed codon bias, with relative synonymous codon usage (RSCU) values greater than 1.0 for at least one codon. Among the most frequently used codons were GCU (Ala, RSCU = 1.52), CAA (Gln, RSCU = 1.50), UAU (Tyr, RSCU = 1.48), CAU (His, RSCU = 1.47), and GGA (Gly, RSCU = 1.41). Additional codons, such as CCU (Pro, RSCU = 1.39), GAU (Asp, RSCU = 1.36), GAA (Glu, RSCU = 1.35), AGA (Arg, RSCU = 1.35), and ACU (Thr, RSCU = 1.35), displayed significant bias. Codon preference patterns indicated that *S*. *glabra* mitochondria favored codons ending in A/U, a tendency already documented in other angiosperm mitochondrial genomes as well (Guo et al., 2024).

### Repeat elements

Simple sequence repeats (SSRs) are important molecular markers that provide insights into genome organization, genetic variation, and evolutionary processes. In the mitochondrial genome of *S*. *glabra*, a total of 399 SSRs were identified (**Figure 3A**). Tetranucleotide repeats were the most abundant with 174 loci, followed by 78 dinucleotide, 66 mononucleotide, 47 trinucleotide, 30 pentanucleotide, and 4 hexanucleotide repeats. Motif analysis revealed that A/T-rich sequences dominated the SSR composition (**Figure 3B**). The A/T motif was the most frequent, occurring 58 times, and AG/CT, AAAG/CTTT, and AT/AT appearing 37, 35, and 34 times, respectively. These results indicated a strong bias toward A/T-rich SSRs, which may be useful for population and evolutionary studies.

**Figure 3:**
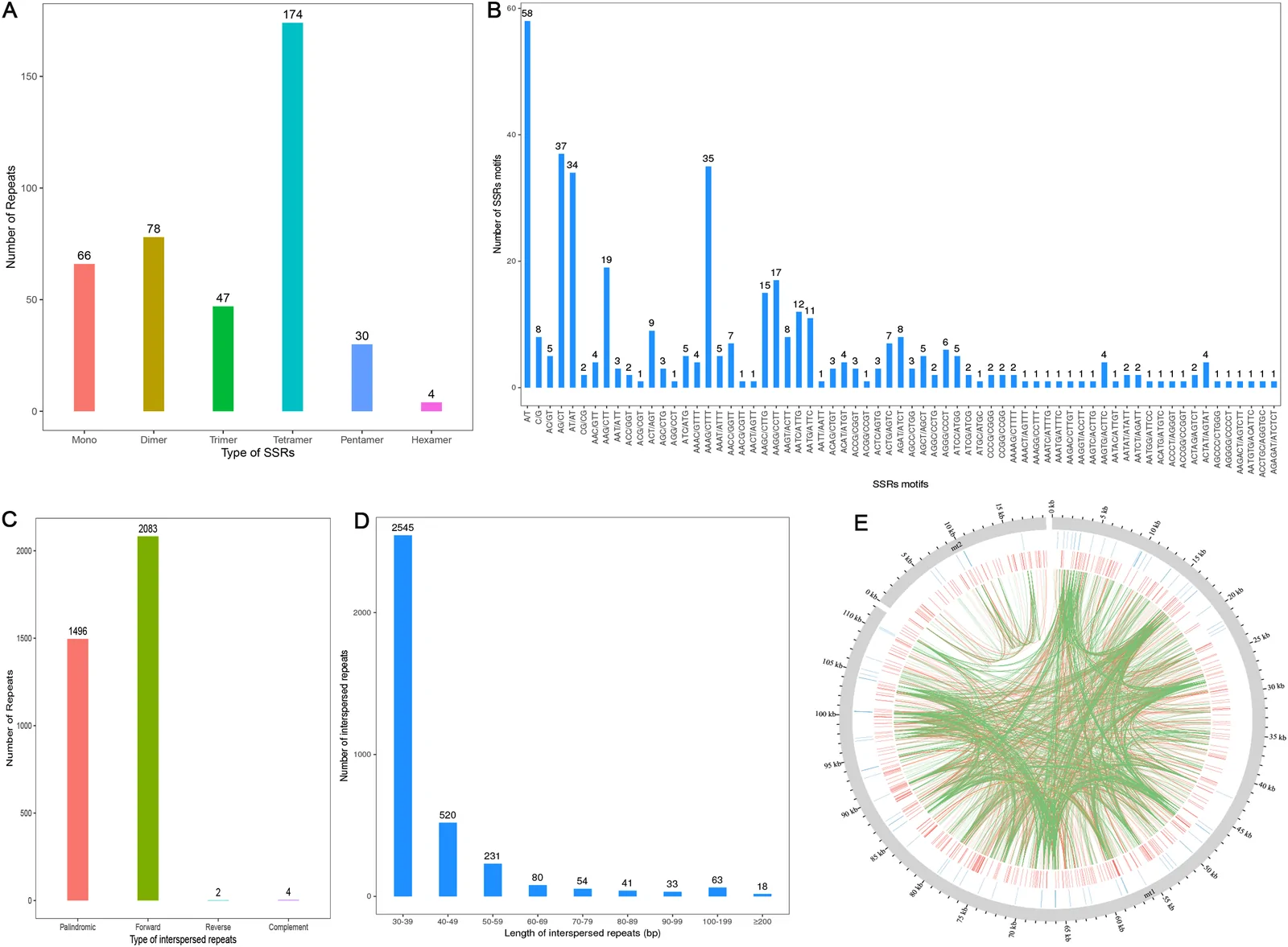
Characterization of simple sequence repeats (SSRs) and interspersed repeats in the *S*. *glabra* mitochondrial genome. **(A)** Distribution of SSRs by repeat unit type. **(B)** Frequency of different SSR motifs. **(C)** Classification of interspersed repeats based on repeat type. **(D)** Distribution of interspersed repeats by length. **(E)** Circos plot of repeat pairs in the mitochondrial genome. Red bars on the outer rings represent repeat regions, while green lines in the interior connect homologous repeat pairs.

Interspersed repeats contribute significantly to genome rearrangements, recombination, and structural plasticity in plant mitochondria. In *S*. *glabra*, a total of 3,585 interspersed repeats were detected. Forward repeats were the most numerous, followed by palindromic repeats. In contrast, only two reverse repeats and four complement repeats were identified (**Figure 3C**). The majority of repeats were short, with 2,545 sequences ranging from 30 to 39 bp (**Figure 3D**). Only 18 repeats exceeded 200 bp in length. Tandem repeat analysis revealed that the longest repeat in the *S*. *glabra* mitogenome reached 76 bp. Most repeats occurred in multiple copies, with an average copy number of approximately 2.76 (**Supplementary Table S4**). Visualization of the large repeats demonstrated extensive repeat-mediated recombination potential throughout the mitochondrial genome (**Figure 3E**), suggesting that interspersed repeats play an essential role in shaping the structural complexity and dynamic evolution of *S*. *glabra* mitochondria.

### RNA editing

RNA editing plays a crucial role in the post-transcriptional regulation of gene expression, especially in the mitochondria, where it is necessary for the optimal functioning of various mitochondrial genes. Initially, computational predictions suggested 891 potential RNA editing sites across the PCGs of the *S*. *glabra* mitogenome (**Supplementary Table S5**). However, recognizing the inherent false-positive rates of predictive tools, we subsequently performed stringent empirical validation using transcriptomic data. This validation strictly authenticated a total of 524 genuine RNA editing sites (**Figure 4**). Among the PCGs, *nad5* exhibited the highest level of RNA editing, with 42 validated sites. This was followed by *nad4*, *nad7*, and *ccmFn*, which also displayed a comparatively large quantity of editing sites, indicating a significant level of post-transcriptional modification. The *atp* and *cox* gene families, including *atp6*, *cox1*, and *cox2*, exhibited moderate RNA editing activity, with *atp6* containing 23 editing sites, *cox1* containing 31 editing sites, and *cox2* containing 19 editing sites. Conversely, the ribosomal protein genes, including *rps7*, *rps11*, and *rps19*, had fewer RNA editing sites ranging from 1 to 5 sites, suggesting a more limited extent of editing in these transcripts.

**Figure 4:**
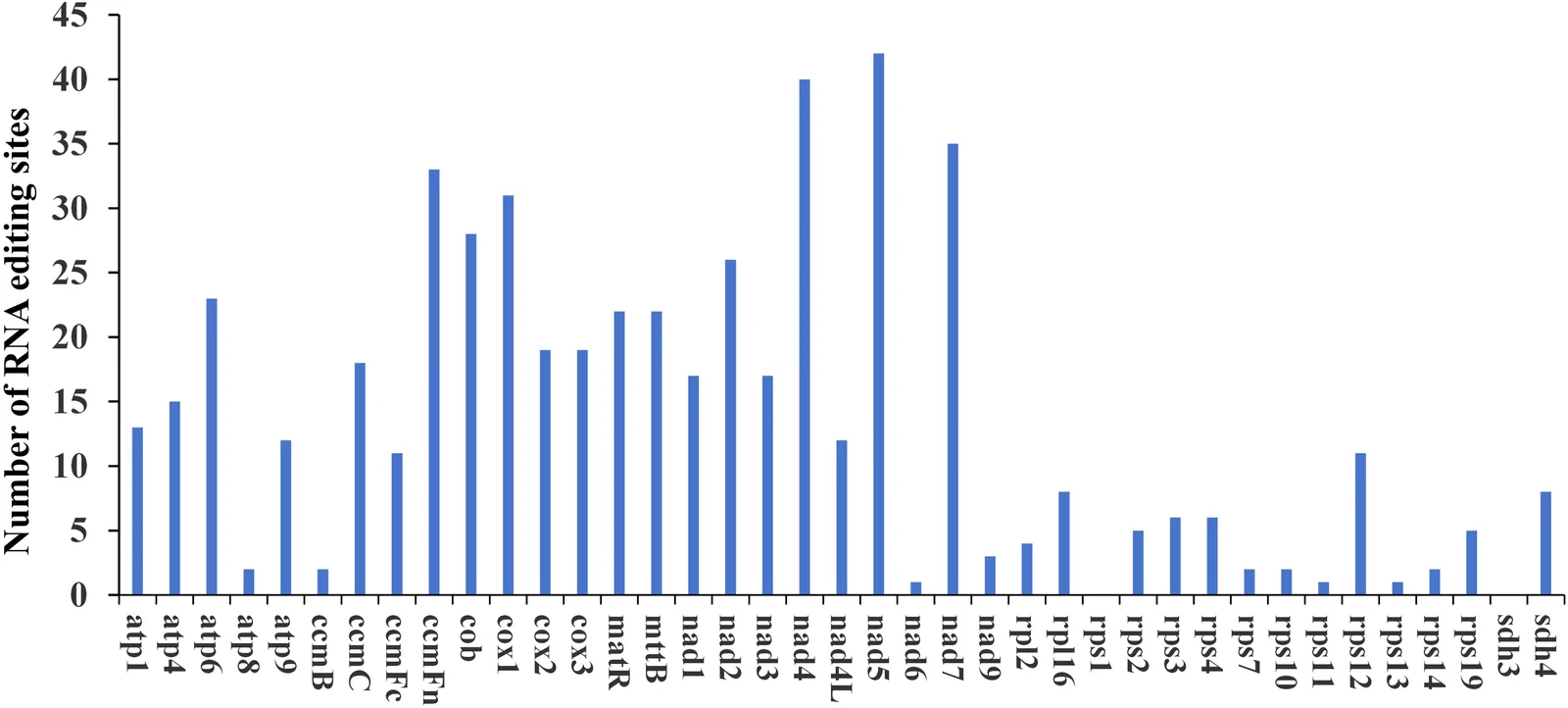
Distribution of validated RNA editing sites across protein-coding genes in the *S*. *glabra* mitochondrial genome. The sites presented here were stringently authenticated through comparative analysis with matched transcriptomic data, excluding false positives from initial computational predictions.

Consistent with typical plant mitogenomes, the majority of editing events were C-to-T conversions, representing 499 of the 524 sites. The most common type of amino acid change was from hydrophilic to hydrophobic residues. Specifically, the conversion of serine to leucine and proline to leucine occurred 107 times, and the transition from proline to leucine occurred 102 times, demonstrating a high frequency across several genes. Furthermore, codon changes leading to termination signals were extremely rare with only 2 such sites identified, which accounted for less than 1% of all editing-induced amino acid changes (**Supplementary Table S6**).

### Genetic diversity and selective pressure analysis

To investigate the evolutionary dynamics of the mitochondrial genome in *S*. *glabra*, nucleotide diversity (Pi) was calculated across PCGs. The results showed considerable variation among different genes, with 4 out of 29 genes exhibited a Pi value higher than 0.05 (**Figure 5**). The highest nucleotide diversity was observed in *atp9* (Pi = 0.071), *nad6* (Pi = 0.068), and *atp1* (Pi = 0.057), suggesting that these genes may have accumulated more polymorphic sites during evolution. In contrast, genes such as *nad7* (Pi = 0.013), *rps14* (Pi = 0.015), and *nad9* (Pi = 0.017) displayed much lower levels of diversity, indicating stronger conservation. Overall, most genes exhibited moderate nucleotide diversity, but several loci showed distinct peaks, reflecting heterogeneous evolutionary constraints within the mitochondrial genome.

**Figure 5:**
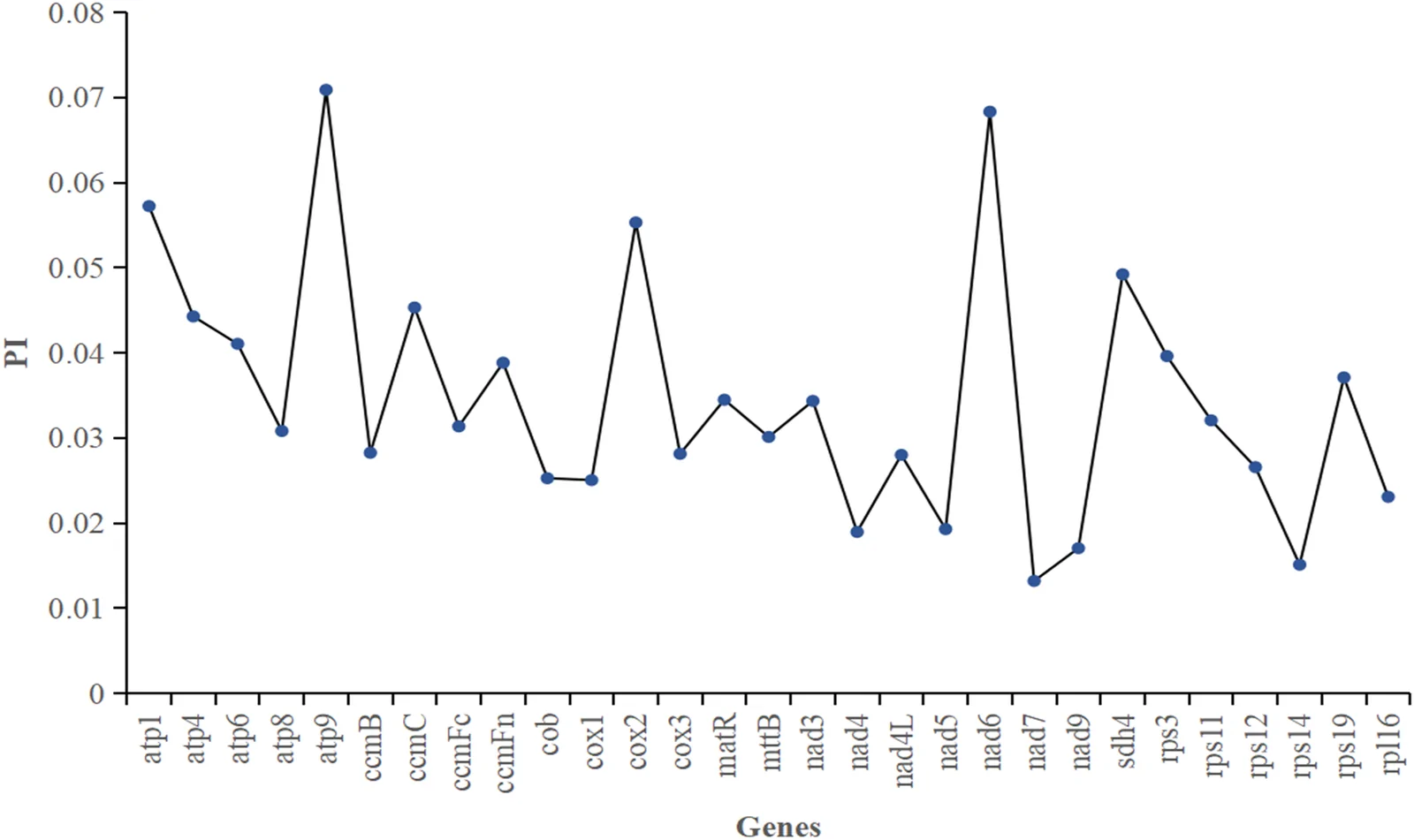
Nucleotide diversity (Pi) values of mitochondrial protein-coding genes in *S*. *glabra*.

To further evaluate the selective forces acting on mitochondrial PCGs, the nonsynonymous to synonymous substitution ratio (Ka/Ks) was calculated by comparing *S*. *glabra* with related species. The majority of genes displayed Ka/Ks ratios below 1 (**Figure 6**), consistent with the action of purifying selection, which was expected for functionally essential mitochondrial genes. However, elevated Ka/Ks ratios were detected in specific genes, such as *atp9*, *cob*, *nad3*, *nad4*, and *rpl16*, in comparison with certain species. Notably, the Ka/Ks ratio of *nad4* exceeded 3 in the comparison with *C*. *praecox*, suggesting potential positive selection. Similarly, *cob* and *rpl16* showed Ka/Ks ratios above 2 in several comparisons, indicating episodes of adaptive evolution. In contrast, genes encoding cytochrome c oxidase such as *cox1*, *cox2*, and *cox3* consistently maintained low Ka/Ks values across all species comparisons, underscoring their highly conserved nature.

**Figure 6:**
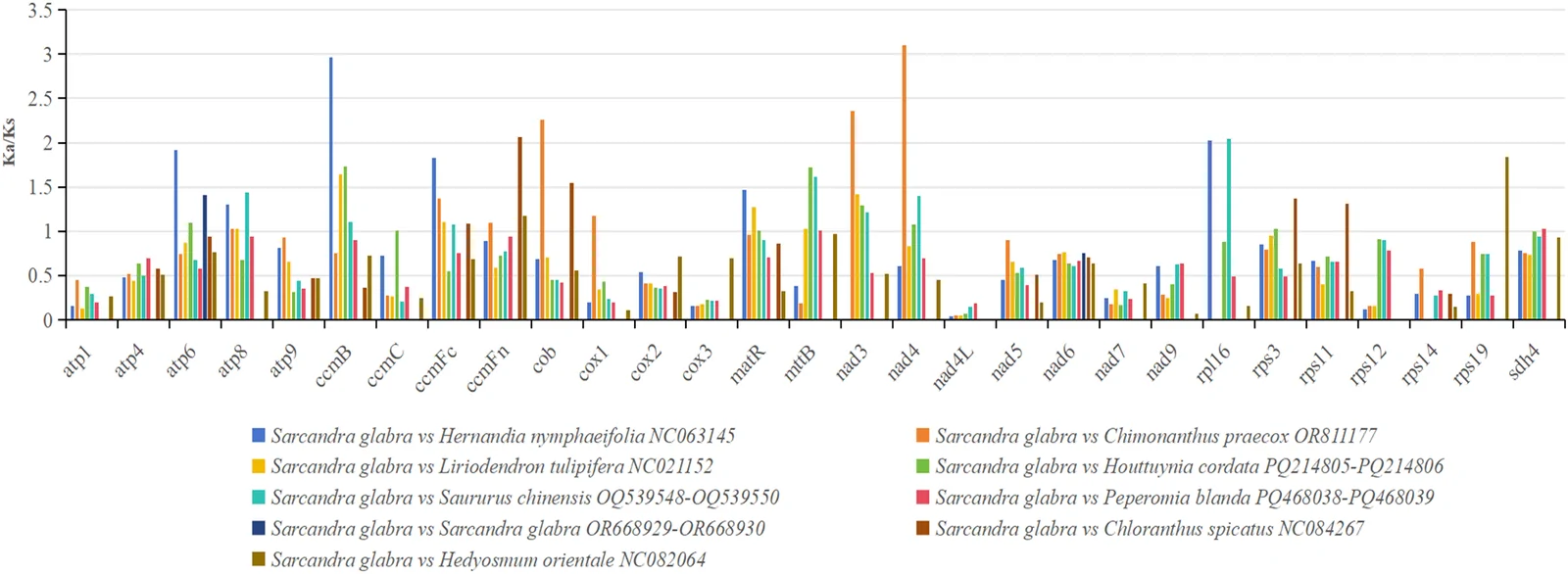
Comparative analysis of non-synonymous (Ka) to synonymous (Ks) substitution ratios (Ka/Ks) of mitochondrial protein-coding genes between *S*. *glabra* and related species.

### Analysis of mitochondrial–plastid transfers

The analysis of MTPTs provides important insights into the evolutionary history and structural dynamics of plant mitochondrial genomes. We identified 29 fragments transferred from the plastome to the mitogenome of *S*. *glabra* (**Figure 7**; **Supplementary Table S7**), with individual fragment lengths ranging from 1,011 bp to 5,648 bp. The cumulative length of these MTPTs was 154,266 bp, accounting for approximately 11.74% of the *S*. *glabra* mitochondrial genome. Notably, all identified MTPTs exceeded 1 kb in length, indicating a bias toward large-scale DNA transfer events. A total of 9 plastid-derived tRNA genes, including *trnL-CAA, trnC-GCA, trnD-GUC, trnR-ACG, trnN-GUU, trnM-CAU, trnQ-UUG, trnI-CAU* and *trnL-UAA*, were identified among these transferred regions, while no complete protein-coding genes were detected within the MTPTs, suggesting that functional gene transfer is limited to RNA elements. In addition, no plastid rRNA genes were detected among the MTPTs, indicating that the transfer events in *S*. *glabra* are largely restricted to tRNA sequences rather than structural RNA or protein-coding genes.

**Figure 7:**
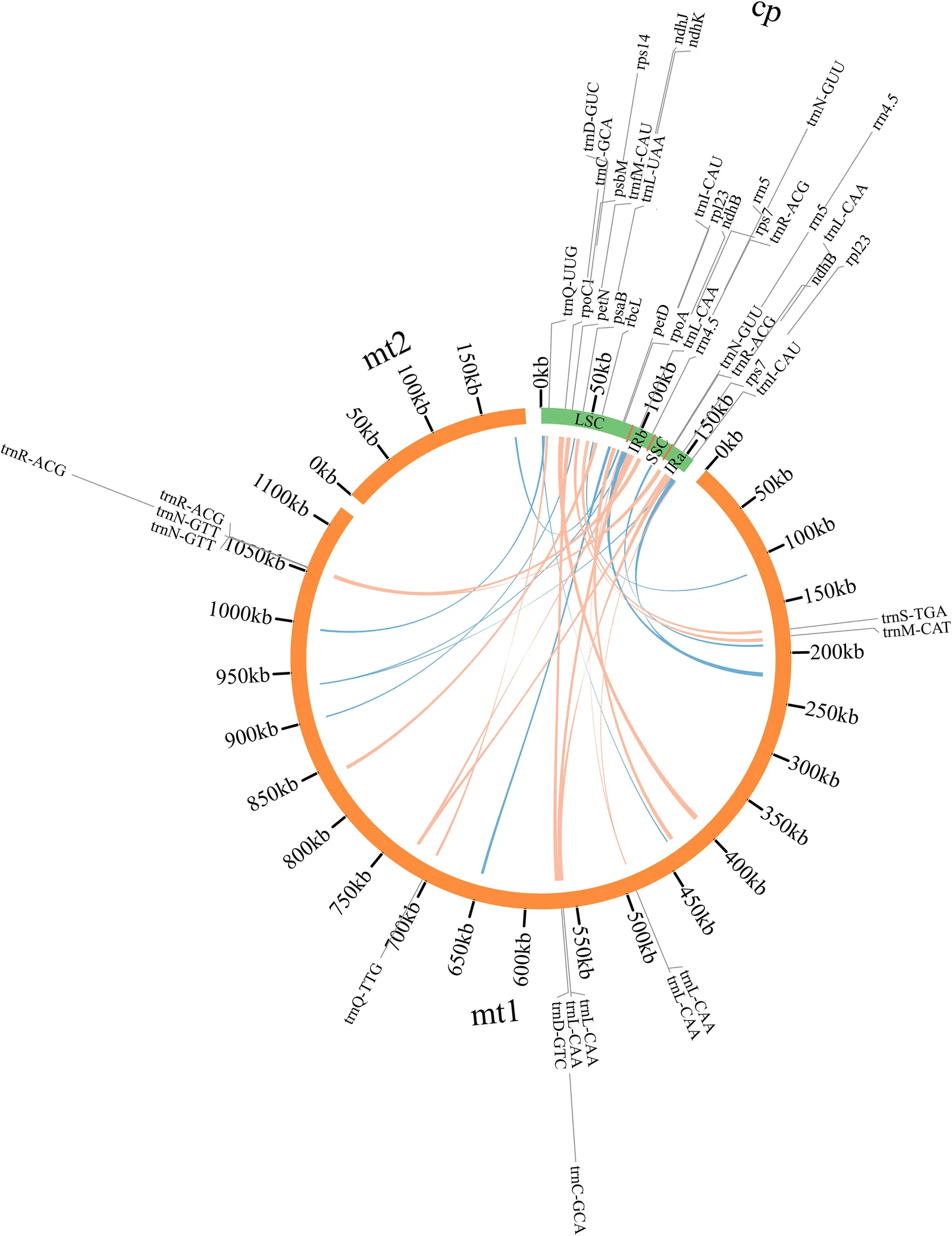
Transfer events of plastid DNA to the mitochondrial genome in *S*. *glabra*. The outer arc represents mtDNA and cpDNA, while the inner arc represents the corresponding transfer mitochondrial plastid DNAs (MTPTs).

### Phylogenetic analysis

To investigate the evolutionary placement of *S*. *glabra*, we constructed a maximum likelihood (ML) tree using complete mitochondrial genome sequences from 21 angiosperm species, representing Lauraceae, Magnoliaceae, Chloranthaceae, and other early-diverging lineages, with *A*. *trichopoda* as the outgroup (**Figure 8**). The resulting topology showed that *S*. *glabra* was unambiguously placed within Chloranthaceae. It clustered with *C*. *spicatus* with a bootstrap support (BS) of 100%, forming a well-supported Chloranthaceae clade. This clade was resolved as sister to *H. orientale*, also with strong support (BS = 100).

**Figure 8:**
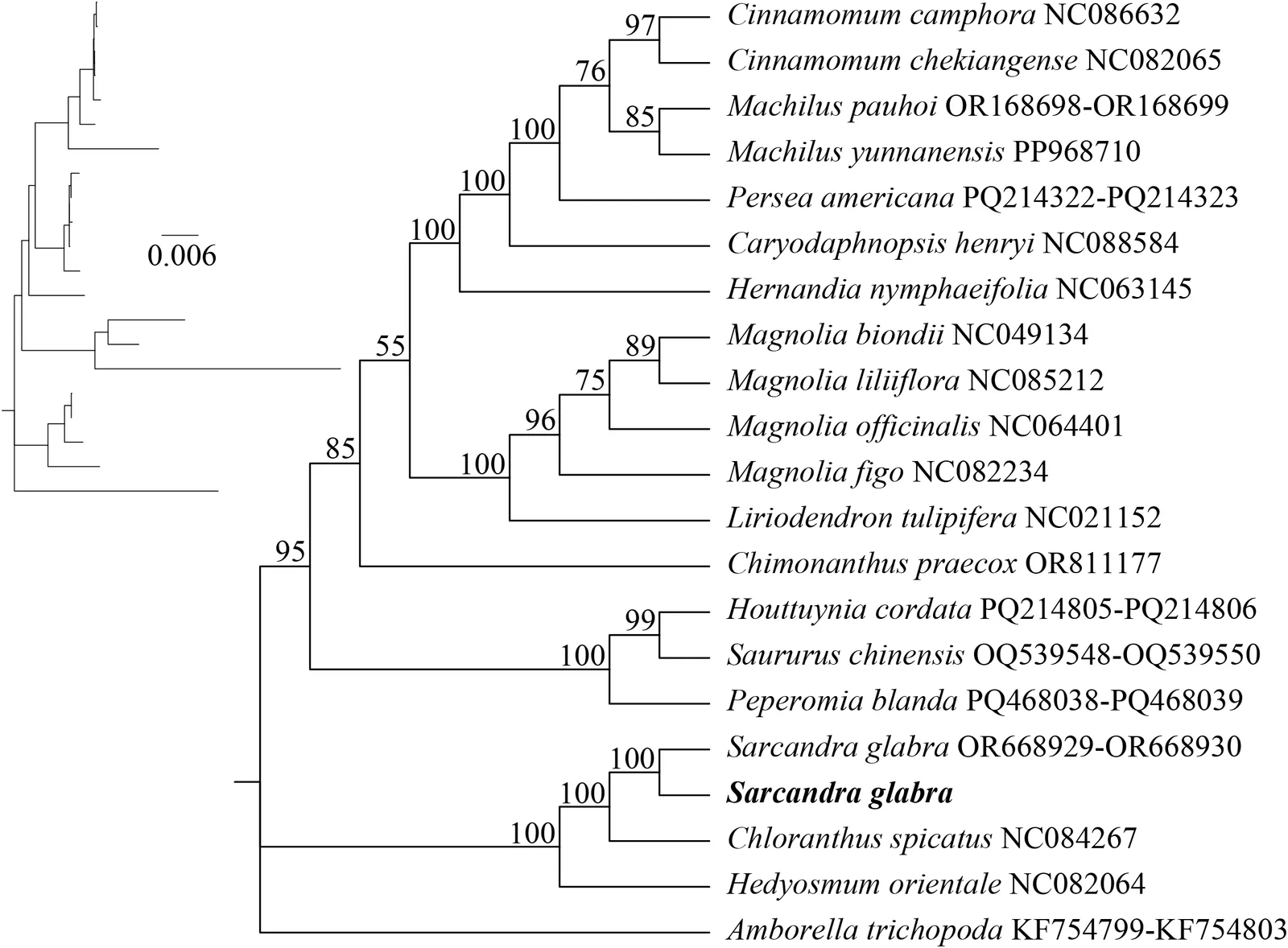
Phylogenetic analysis of *S*. *glabra* and related species based on mitochondrial genome sequences. A maximum likelihood (ML) phylogenetic tree was constructed using concatenated mitochondrial protein-coding gene sequences from *S*. *glabra* and other angiosperm species. Bootstrap support values (out of 100) are indicated at the nodes.

Within Lauraceae, *C*. *camphora* and *C. chekiangense* grouped together (BS = 97) and further clustered with *M*. *pauhoi* and *M. yunnanensis* (BS = 85), while *P*. *americana* and *C*. *henryi* branched nearby. Magnoliaceae species formed a distinct monophyletic group with high support values: *M*. *biondii* and *M. liliiflora* grouped with 89% support, which further grouped with *M. officinalis* and *M. figo*. *L*. *tulipifera* was clearly separated but remained within the Magnoliaceae clade (BS = 100).

To directly address the utility of the identified hypervariable genes as specific phylogenetic markers for Chloranthaceae, we constructed and compared independent ML trees using six different datasets: all PCGs, conserved PCGs, concatenated hypervariable genes (*atp1*, *atp9*, *nad6*), and individual alignments of *atp1*, *atp9*, and *nad6* (**Supplementary Figure S3**). Consistent with the robust topology described above, both the whole-PCG dataset and the conserved PCG dataset produced identical, highly resolved topologies with maximum bootstrap support (BS = 100) across all major nodes within Chloranthaceae. In stark contrast, trees based on the hypervariable genes displayed significant topological incongruence and phylogenetic noise. While the single *atp1* gene tree recovered the correct taxonomy with moderate support, the concatenated hypervariable dataset and the individual *atp9* tree produced conflicting topologies, erroneously grouping *Sarcandra* with *Hedyosmum* instead of *Chloranthus*. Furthermore, the *nad6* tree failed to recover the monophyly of the two *S*. *glabra* accessions. These comparative results indicate that while high nucleotide diversity (Pi) exists in these specific mitochondrial genes, it introduces substantial phylogenetic noise and does not necessarily translate to reliable phylogenetic signals for reconstructing relationships within Chloranthaceae.

## Discussion

The mitochondrial genome is essential for plant cellular function, acting as the energy metabolic powerhouse and offering critical insights into genome evolution and phylogenetic relationships (Sloan, 2013; Gualberto and Newton, 2017). As an early-diverging angiosperm, *S*. *glabra* offers an important window into the evolutionary history of plant mitochondrial genomes (Qiu et al., 2007; Dong et al., 2018). In this study, we systematically investigated the mitochondrial genome of *S*. *glabra*, integrating analyses of genome architecture, codon usage, RNA editing, genetic diversity, mitochondrial–plastid transfers (MTPTs), repeat elements, and phylogenetic placement. This comprehensive analysis allowed us to identify both conserved and lineage-specific characteristics, illuminating the evolutionary processes of Chloranthaceae and early angiosperms in their entirety (Guo et al., 2020; Mower, 2020).

### Structural plasticity and organellar DNA exchange

The *S*. *glabra* mitogenome shows a remarkable degree of structural plasticity, consisting of both linear and circular molecules. Such multibranched architectures have been reported in other angiosperms, including *Silene* and *Cucumis*, and are increasingly recognized as a hallmark of dynamic mitochondrial genome evolution (Sloan, 2013; Kozik et al., 2019). The presence of both structural forms in *S*. *glabra* highlights the ability of plant mitochondria to maintain genomic integrity despite extensive rearrangements and complex physical conformations.

Repeats appear to be a major driver of this structural diversity. We identified 3,585 interspersed repeats, dominated by short forward and palindromic motifs, along with 399 simple sequence repeats (SSRs), most of which were A/T-rich. These features align with observations in *Arabidopsis thaliana*, *Vitis vinifera*, and *Oryza sativa*, where short repeats and A/T-rich microsatellites dominate mitochondrial repeat landscapes (Arrieta-Montiel and Mackenzie, 2011; Kuntal and Sharma, 2011). However, the presence of relatively long repeats (>200 bp) in *S*. *glabra* is notable. Such long repeats can mediate homologous recombination, generating subgenomic circles and contributing to the multibranched conformation we observed. This suggests that recombination-driven rearrangements play a central role in shaping *S*. *glabra* mitogenome architecture and could have important consequences for genome stability and transmission across generations.

A further layer of complexity comes from the integration of plastid-derived DNA. We identified 29 MTPT fragments, totaling ~154 kb, or ~11.7% of the mitochondrial genome. While this level of plastid contribution is comparable to or higher than in many other angiosperms (Mower et al., 2012), *S*. *glabra* shows a striking pattern: only tRNA genes were retained, with no plastid protein-coding or rRNA genes preserved. In contrast, other species, such as *Nicotiana tabacum* and *Arabidopsis thaliana*, frequently maintain plastid-derived rRNA and even functional protein-coding sequences within their mitogenomes (Adams and Palmer, 2003). This suggests that *S*. *glabra* has undergone selective retention of specific plastid elements, possibly linked to its translational machinery. The enrichment of plastid-derived tRNAs could supplement the native mitochondrial tRNA pool, thereby facilitating efficient protein synthesis, or may simply reflect historical recombination biases favoring the insertion and persistence of short, stable non-coding elements. Collectively, the repeat-driven recombination dynamics and plastid-to-mitochondrion DNA transfer highlight the structural fluidity of *S*. *glabra* mitochondria, revealing both conserved mechanisms of genome remodeling and lineage-specific evolutionary trajectories.

### Evolutionary dynamics of the *S*. *glabra* mitochondrial genome

Despite this structural variability, the functional core of the *S*. *glabra* mitogenome remains highly conserved. As in most angiosperms, the majority of protein-coding genes showed low Ka/Ks ratios, consistent with strong purifying selection maintaining essential functions in respiration and energy metabolism (Smith and Keeling, 2015; Li X. et al., 2025). Nevertheless, several genes exhibited signatures of accelerated evolution. For instance, *atp9*, *nad4*, and *rpl16* showed elevated nucleotide diversity and Ka/Ks ratios suggestive of relaxed or positive selection. Similar patterns have been observed in other early-diverging angiosperms such as *L*. *tulipifera* (Park et al., 2015) and *H*. *cordata* (Huang et al., 2025), particularly among genes encoding components of the electron transport chain. This may reflect lineage-specific adaptations to distinct ecological niches, where changes in mitochondrial respiratory proteins could optimize energy metabolism under particular environmental conditions. Specifically, given that *S*. *glabra* is a shade-tolerant evergreen shrub typically inhabiting forest understories, the positive selection observed in genes encoding crucial electron transport chain components may represent a vital evolutionary strategy. These adaptive mutations could enhance the efficiency of oxidative phosphorylation, ensuring sufficient cellular energy production in low-light environments where photosynthetic energy generation is severely restricted.

RNA editing adds another layer of evolutionary and functional complexity, acting as a post-transcriptional buffer against genomic mutations (Small et al., 2020). Following strict transcriptomic validation, we confirmed 524 genuine editing sites in the *S*. *glabra* mitogenome. The vast majority of these are C-to-U conversions, reflecting a canonical pattern universally retained across plant mitochondria (Takenaka et al., 2013). The retention of over five hundred functional editing sites in *S*. *glabra* strongly aligns with genomic patterns observed in other early-diverging angiosperms, such as members of the Magnoliidae (Richardson et al., 2013) and basal ANITA grade (Rice et al., 2013). Unlike some highly derived angiosperm lineages that have drastically reduced their RNA editing machineries through mechanisms like retrotranscription (Fujii and Small, 2011), basal lineages like *S*. *glabra* heavily rely on this extensive post-transcriptional regulation to correct detrimental genomic mutations and maintain transcript viability. Many of these edits resulted in nonsynonymous substitutions, frequently altering amino acid hydrophobicity and potentially enhancing protein stability within the inner mitochondrial membrane. Notably, the high editing frequency in *nad* genes suggests a key role for post-transcriptional modification in tuning NADH dehydrogenase complex activity, a central component of the electron transport chain. For an understory species like *S*. *glabra*, maintaining such a flexible, RNA-mediated regulatory mechanism may provide vital metabolic adaptability, allowing the plant to rapidly adjust its energy metabolism in response to fluctuating environmental stresses such as changing light intensities or temperature shifts in its natural habitat (Zhu et al., 2014). The occasional generation of stop codons, though rare, may have regulatory significance, producing truncated proteins or triggering nonsense-mediated mRNA decay. Such events highlight the intricate post-transcriptional regulation of mitochondrial gene expression and underscore the dynamic interplay between RNA-level modifications and protein-level function.

Codon usage bias in *S*. *glabra* further illustrates selective forces acting at the translational level. While the strong preference for A/U-ending codons is a characteristic feature shared with many other angiosperm mitogenomes (Meng and Xia, 2009), our comprehensive analysis reveals that this bias is not solely the result of directional mutation. The ENC-plot analysis demonstrated that the majority of protein-coding genes cluster well below the expected curve (Wright, 1990), providing compelling evidence that natural selection plays a dominant role in shaping these codon usage patterns, a phenomenon increasingly recognized in the evolution of plant mitogenomes (Hao et al., 2025). This conclusion is further corroborated by the PR2-bias analysis (Sueoka, 1995), which revealed a distinct preference for Cytosine over Guanine at the third codon position, deviating from the equilibrium expected under pure mutation pressure. Collectively, these findings suggest that while mutational biases may establish the baseline nucleotide composition, natural selection acts as the primary driving force, likely reflecting an adaptive optimization of translation efficiency and accuracy in the *S*. *glabra* mitochondria. Together, the combination of purifying selection, gene-specific positive selection, extensive RNA editing, and codon usage bias paints a picture of a mitochondrial genome that is both functionally constrained and evolutionarily innovative.

### Phylogenetic implications for early angiosperm evolution

Beyond its structural and functional characteristics, the *S*. *glabra* mitochondrial genome provides valuable phylogenetic insights into early-diverging angiosperms. Our phylogenetic analyses, relying on conserved mitochondrial genes, placed *S*. *glabra* firmly within Chloranthaceae, clustering with *C*. *spicatus* and resolving the family as sister to *H*. *orientale*. These results are consistent with plastid and nuclear phylogenomic studies (Qiu et al., 2007; Li et al., 2019) and reinforce the monophyly of Chloranthaceae as a distinct early-diverging lineage. The congruence of mitochondrial, plastid, and nuclear signals strengthens confidence in this placement, suggesting that even structurally complex mitochondrial genomes retain robust phylogenetic utility (Barkman et al., 2000).

Importantly, the placement of Chloranthaceae near the base of the angiosperm tree, in close association with magnoliids, has significant implications for our understanding of early angiosperm diversification. As an evergreen shrub with a disjunct distribution in East Asia and Southeast Asia, *S*. *glabra* may also hold clues to the historical biogeography of early angiosperms. Comparative mitogenomic studies across Chloranthaceae could reveal whether the patterns observed here—such as high repeat-mediated recombination and plastid-derived tRNA enrichment—are lineage-wide characteristics or unique to *S*. *glabra*.

To complement these organellar perspectives, publicly available transcriptomic repositories provide a valuable resource for expanding our evolutionary understanding of *S. glabra* and related taxa. Researchers can computationally assemble these raw sequencing datasets and subsequently apply the BUSCO pipeline to extract conserved single copy nuclear orthologs (Wong and Peakall, 2022). This data mining strategy offers a highly efficient method to construct robust nuclear phylogenies without additional sequencing costs (Arreguin et al., 2025). Integrating these biparentally inherited nuclear markers is essential for identifying complex evolutionary events such as ancient hybridization or incomplete lineage sorting. Comparing these nuclear evolutionary trajectories with our maternally inherited mitogenome assembly provides a comprehensive framework to definitively resolve cytonuclear discordance within the Chloranthaceae family.

Building upon this framework, recent studies have already successfully utilized nuclear data to resolve the phylogenetic position of Chloranthaceae. For instance Zeng et al. (2014) employed 59 conserved low copy nuclear genes to establish a highly supported topology for Mesangiospermae (Zeng et al., 2014). Their nuclear phylogeny accurately placed *S*. *glabra* and resolved deep angiosperm relationships. Similarly Li et al. (2025) utilized nuclear ribosomal DNA alongside plastomes to reconstruct the evolutionary history of Chloranthaceae (Li PW. et al., 2025). They observed strong congruence between nuclear and plastid trees at the intergeneric level. However they also identified specific cytonuclear discordance within the genus Hedyosmum. Cross referencing our mitogenome assembly with these nuclear phylogenies bridges a critical gap. Combining our high quality mitochondrial data with these established nuclear markers provides a comprehensive framework for resolving the evolutionary dynamics of *S*. *glabra*.

Crucially, our targeted assessment of phylogenetic markers reveals the complex evolutionary dynamics of mitochondrial genes. It is a common assumption in molecular systematics that genetic regions with high nucleotide diversity (Pi) provide better resolution for closely related taxa. However, our comparative analyses using different datasets (**Supplementary Figure S3**) clearly demonstrate that this principle does not universally apply to the *S*. *glabra* mitogenome. While the concatenated conserved protein-coding genes (PCGs) yielded a highly robust topology (BS = 100), the hypervariable genes (*nad6* and *atp9*), despite their elevated mutation rates, introduced substantial phylogenetic noise and topological conflicts. From an evolutionary perspective, this incongruence may be driven by substitution saturation and homoplasy—where rapid, independent mutations at the same loci obscure true phylogenetic signals (Knoop, 2004; Sloan et al., 2012). Furthermore, plant mitochondrial genes are subject to extensive and lineage-specific RNA editing, which can alter evolutionary constraints at the DNA level and complicate phylogenetic inferences if not carefully accounted for (Gualberto and Newton, 2017). Therefore, we strongly recommend that future evolutionary studies in Chloranthaceae prioritize whole-mitogenome conserved datasets over individual high-Pi genes to avoid misleading taxonomic conclusions. Our findings highlight a paradox of plant mitochondrial genomes: despite being among the most structurally dynamic and idiosyncratic genomes in eukaryotes, they continue to provide coherent phylogenetic signals at deep evolutionary scales. In *S*. *glabra*, the combination of structural fluidity and phylogenetic robustness illustrates how the plant mitochondrial genome functions simultaneously as a molecular laboratory of recombination and a reliable archive of evolutionary history. Nevertheless, it is important to acknowledge that our current structural and evolutionary inferences are based on a single individual and rely primarily on computational genomic data. Therefore, future studies incorporating population-level sampling will be necessary to fully assess intraspecific structural variations and pan-mitogenome dynamics. Additionally, experimental validations—such as transcriptomic profiling or biochemical assays—are required to definitively confirm the physiological and ecological impacts of the identified RNA editing sites and positively selected genes in adapting to understory environments. By addressing these aspects alongside expanded taxon sampling across early angiosperms, future research can more fully unravel the evolutionary forces shaping this complex genome.

## Conclusion

This study provides the first comprehensive mitochondrial genome of *S*. *glabra*, elucidating its genomic structure and evolutionary characteristics. The mitogenome had a multi-branched structure and contained vital genes involved in energy production and RNA maturation, emphasizing its functional complexity. The identification of codon biases and substantial RNA editing sites highlighted the evolutionary forces shaping its mitochondrial genome, while genetic diversity and selection pressure analyses revealed both conserved and adaptive genomic regions. The presence of simple sequence repeats and interspersed repeats suggested an underlying structural plasticity, potentially facilitating mitogenomic rearrangements and evolution in *S*. *glabra*. By placing *S*. *glabra* within the Chloranthaceae, this research deepened our understanding of plant mitochondrial evolution, offering insights into gene transfer, adaptive processes, and the genomic architecture of early-diverging angiosperms. These results provided a basis for future comparative genomics and evolutionary studies in plant mitochondrial biology.

## Data Availability

The datasets presented in this study can be found in online repositories. The names of the repository/repositories and accession number(s) can be found below: https://www.ncbi.nlm.nih.gov/genbank/, PX754771 https://www.ncbi.nlm.nih.gov/genbank/, PX754772.

## Supplementary material

Supplementary Table 1 Gene annotation of the *S*. *glabra* mitochondrial genome.

Supplementary Table 2 Summary of genomic features of the two mitochondrial molecules (Molecule 1 and Molecule 2) in *S*. *glabra.*

Supplementary Table 3 Number of codon usage of protein-coding genes in the *S*. *glabra* mitochondrial genome.

Supplementary Table 4 Tandem repeats in the *S*. *glabra* mitochondrial genome.

Supplementary Table 5 The type of RNA editing in the *S*. *glabra* mitochondrial genome.

Supplementary Table 6 List of RNA-seq validated RNA editing sites in the protein-coding genes of the *S. glabra* mitogenome.

Supplementary Table 7 Homologous sequence of mtDNA and cpDNA in *S*. *glabra.*

Supplementary Figure 1 *S*. *glabra* plant with mature fruits.

Supplementary Figure 2 Sequencing depth distribution across the *S*. *glabra* mitogenomes.

Supplementary Figure 3 Assessment of phylogenetic marker utility in the *S*. *glabra* mitogenome. Maximum Likelihood (ML) phylogenetic trees were reconstructed using different datasets: **(A)** all protein-coding genes (PCGs), **(B)** conserved PCGs, **(C)** concatenated hypervariable genes (*atp1*, *atp9*, *nad6*), **(D)** *atp1* gene, **(E)** *atp9* gene, and **(F)** *nad6* gene.
